# Combination of Oncolytic Measles Virus Armed With BNiP3, a Pro-apoptotic Gene and Paclitaxel Induces Breast Cancer Cell Death

**DOI:** 10.3389/fonc.2018.00676

**Published:** 2019-01-15

**Authors:** Geetanjali Lal, Maitreyi S. Rajala

**Affiliations:** School of Biotechnology, Jawaharlal Nehru University, New Delhi, India

**Keywords:** triple negative breast cancer/TNBC, oncolytic virus, measles virus, BNiP3, paclitaxel, combination therapy, apoptosis, H2 compound

## Abstract

Breast cancer is one of the major causes of cancer related mortality in women worldwide. Limitations of conventional anti-cancer therapies such as severe systemic side effects, narrow therapeutic index, non-specificity, and non-availability of drugs for all types of cancers has resulted in the development of various novel and targeted approaches. The use of viruses as oncolytic agents has gained momentum for the development of an efficient therapeutic platform. In this study, we have developed recombinant measles virus armed with BNiP3, a pro-apoptotic gene of human origin, as an oncolytic agent, and have demonstrated its ability to induce apoptosis in breast cancer cells *in vitro*. Studies have demonstrated the potential of using oncolytic viruses in combination with conventional therapies as an efficient anti-cancer regimen. We also have explored the synergistic potential of this virus in combination with paclitaxel, and a hydrazone derivative, H2 compound as an anti-cancer agent. MCF-7 and MDA-MB-231, human breast cancer cell lines were used for *in vitro* studies to evaluate toxic effects of armed virus, rMV-BNiP3 both as a standalone therapy and in combination with paclitaxel or H2 compound, a hydrazone derivative. Generation of armed virus was confirmed by detecting the viral transcript and protein expression, while its oncolytic potential by cell viability assays. Induction of apoptosis was demonstrated by fluorescence based caspase 3 activity and flow cytometry based Annexin V/PI staining. In the current study we have demonstrated the successful generation of an oncolytic measles virus armed with BNiP3 (rMV-BNiP3) and the induction of toxic effects in rMV-BNiP3 infected cells with a curious bias toward MDA-MB-231 cells as compared to MCF-7. Infection of breast cancer cells with rMV-BNiP3 caused induction of cell death, but the combination of rMV-BNiP3 with sub-lethal doses of both paclitaxel and H2 lowered the overall viability of cancer cells. As triple negative breast tumors are highly aggressive and resistant subtype of breast cancer with poor prognosis, comparative sensitivity of MDA-MB-231 cells toward this virus may potentially be used to develop a targeted therapy against triple negative breast cancer.

## Introduction

Breast cancer is the second leading cause of cancer related deaths worldwide and amongst the most prevalent in women (1). Yearly 1.7 million new breast cancer cases are diagnosed globally of which 60% deaths occur in developing countries (2). The death rate of breast cancer has dropped in recent years; nonetheless it remains relatively higher in developing countries due to delayed diagnosis and treatment. There exists differentiation of prognosis and treatment response based on the subtype of disease with hormone-refractory triple negative breast cancer (TNBC) showing greater metastasis, resistance, and mortality (3, 4). Currently available treatments; tamoxifen; and trastuzumab based therapies are targeted toward estrogen receptor positive (ER+) and Her2/neu positive cancers, respectively. TNBC, being negative for estrogen receptor (ER), progesterone receptor (PR), and human epidermal growth factor (Her2) does not respond to these therapies and presents itself as the most aggressive subtype. Surgery and chemotherapy are the two treatment modalities for TNBC patients but the prognosis usually remains poor. TNBC and ERBB2 negative breast cancer leads to brain metastasis in ~20–50% of cases (5). Thus, therapeutic strategies are being developed for TNBC treatment involving multimodal application of therapies in synergistic combination.

Recent studies with oncolytic viruses have shown their efficacy toward the development of a targeted anti-cancer approach. There are many viruses with natural oncolytic activity such as Reovirus, Newcastle disease virus, vesicular stomatitis virus, moloneyleukemia virus, parvovirus, mumps virus etc. Some viruses such as measles, adenoviruses, HSV, VSV, and vaccinia virus can be adapted for tumor specificity by repeated laboratory cultures giving rise to low virulent strains but most of the times exhibit tumor tropism (6). Currently many viruses have been manipulated and retargeted toward cancer cells with modified receptors, increasing the safety of virus by attenuating its pathogenicity without disrupting its replication ability. These natural or adapted tumor-tropic viruses have emerged as potential oncolytic agents and are undergoing pre-clinical and clinical studies for their cytotoxic effects against various cancers. Amongst many viral candidates, vaccine strain of measles virus has been one of the most promising oncolytic virotherapeutic platforms with population wide safety and ease of retargeting toward tumor-specific surface antigens.

Measles virus is an enveloped virus with non-segmented negative sense RNA genome and uses SLAM receptor for internalization into the host. Whereas, the vaccine strain of measles virus uses CD46 receptor which is often overexpressed in many tumors including breast carcinoma. However, vaccine strains retain their ability to bind to SLAM receptor; its ability to lyse primary mantle cell lymphoma cells in the absence of CD46 has been demonstrated (7). Nectin-4 is another receptor being used by measles virus which is commonly expressed in respiratory epithelial cells. Measles virus mediated lysis of pancreatic cancer cells with nectin-4 expression was also reported (8). Retargeting of measles virus can also be achieved through modification of its surface proteins; hemagglutinin (9), and fusion protein (10). Besides its affinity to various receptors for internalization; impaired immune response of cancer cells is another factor which contributes to the materialization of measles virus as an efficient oncolytic agent (11). Currently measles virus based oncolytics are in phase I and II clinical trials for treatment of glioblastoma, ovarian cancer, multiple myeloma, and mesothelioma (12).

Oncolytic virotherapy relies on cancer specific replication of virus triggering tumor cell death by a number of mechanisms, including direct lysis, induction of apoptosis, expression of toxic proteins, autophagy, and induction of anti-tumoral immunity (13, 14). These features of oncolytic viruses can further be enhanced by arming with foreign genes encoding for antitumor activity. Insertion of a gene encoding cytokine granulocyte macrophage colony stimulating factor (GM-CSF) reduced the colon adenocarcinoma in mouse model following intratumoral administration of MV-GM-CSF (15). Measles virus armed with suicide gene, super cytosine deaminase (SCD) induced tumor cell death in mouse xenograft model of hepatocellular carcinoma (16). Since viruses differ in their mode of action from conventional therapies and are amenable to modifications involving targeting and arming, they can be developed as drug of choice to induce cell death in chemotherapy and radiotherapy resistant cancers.

In the current study, we have developed a measles virus armed with BNiP3, pro-apoptotic gene of human origin (rMV-BNiP3) as a therapeutic agent for treatment of breast carcinoma both as a monotherapy and a component of combination therapy based on chemotherapy. BNiP3, Bcl-2/adenovirus E1B1 19 kDa interacting protein 3, is a hypoxia responsive protein via HIFα signaling, generally involved in autophagy and mitophagy. Many studies have elucidated the duality of BNiP3's role as a cancer-type dependant tumor repressor or promoter (5, 17–19). As a component of viral genome, we hypothesized that the presence of BNiP3 in addition to oncolytic nature of Edmonston strain of measles virus will cause induction of apoptosis, and cell death in breast cancer cells. Furthermore, oncolytic activity of rMV-BNiP3 was evaluated in the presence of paclitaxel, chemotherapeutic drug approved for the treatment of various cancers including breast carcinoma. Paclitaxel acts as a stabilizer of microtubules causing mitotic arrest due to inhibition of spindle fiber dissociation leading to cell death. There have been pre-clinical and clinical studies involving combination of paclitaxel with oncolytic virus such as MG1 maraba virus (20) in murine breast carcinoma, herpes virus (21) in mice model of anaplastic thyroid cancer, vaccinia virus (22) in mice model of colorectal cancer and reovirus (23) in phase I/II studies involving patients with incurable, metastatic head and neck carcinoma. We wanted to observe the effectivity of rMV-BNiP3 with paclitaxel. In addition to the use of an approved chemotherapeutic agent; we have used a hydrazone derivative, H2, for the combinatorial approach with rMV-BNiP3 to ascertain its anti-tumor activity. Our preliminary results demonstrated enhanced cell death in TNBC cell line, MDA-MB-231 over MCF-7 cells infected with rMV-BNiP3 combined with drugs. Current study focuses on the development of an armed measles virus as a viable oncolytic agent against breast cancer especially TNBC. Here we have demonstrated the compatibility of this virus with both an approved chemotherapeutic agent and H2 compound to further establish the role it can play as a targeted component of a multimodal anti-cancer therapy against breast cancer.

## Materials and Methods

### Cell Lines and Cultures

HEK293, human embryonic kidney epithelium; MCF-7 and MDA-MB-231, human breast adenocarcinoma cell lines (NCCS, Pune, India) were propagated in Dulbecco's Modified Eagle Medium (DMEM) whereas Vero, monkey kidney epithelium cell line was grown in Modified Eagle Medium (MEM) supplemented with 10% fetal bovine serum (FBS) and maintained at 37°C in a humidified atmosphere with 5% CO_2_. All the culture reagents were purchased from HiMedia.

### Generation of Recombinant Clones

Recombinant clones for measles virus Nucleoprotein (N), capsid protein and Phosphoprotein (P), co-factor for viral RNA dependent RNA polymerase were generated from clinical isolate of measles virus obtained from AIIMS, New Delhi. Total RNA was isolated using Trizol (Biobasic, USA) method from infected Vero cells, reverse transcribed and cDNA was amplified using gene specific primers for N; FP:5′ATATGAATTCACCATGGCCACACTTTTGAGGAG3′, RP:5′ATATCTCGAGCTAGTCTAGAAGATCTCTGTC3′ and for P; FP:5′ATATGAATTCACCATGGCAGAAGAGCAGGCAC3′, RP: 5′ATATCTCGAGCTACTTCATTATTATTATCTTCATC3′. Amplicons were purified and cloned into eukaryotic expression vector pcDNA3.1(+) between *Eco*RI and *Xho*I restriction sites. Recombinant clone for T7 RNA polymerase (T7) was similarly generated using cDNA prepared from RNA isolated from BL21pLysS culture. Complimentary DNA was amplified using gene specific primers for T7; FP: 5′ATATGAATTCACCATGAACACGATTAACATCGC3′, RP: 5′ATATCTCGAGTTACGCAACGCGAAGTCCG3′, and cloned into pcDNA3.1(+) between *Eco*RI and *Xho*I. For the rescue of virus, recombinant clone for viral Large polymerase (L), an RNA dependant RNA polymerase was generated with gene specific primers; FP: 5′ATATGAATTCACCATGGACTCGCTATCTGTCAAC3′, RP:5′ATATCTCGAGTTAATCCTTAATCAGAGCGCTG3′. Amplified gene was cloned into pcDNA3.1(+) between *Bam*HI and *Not*I restriction sites. All the clones were confirmed by sequencing and L gene was confirmed end-to-end with 4 sets of nested primers.

### Generation of Reporter and Armed Recombinant Measles Virus

The plasmid vector, p(+)MV-NSE-FlagP-M502-3p, encoding full length anti-genome sequence of measles virus Edmonston strain was a gift from Branka Horvat (Addgene plasmid #58748) and was used to generate desired recombinant measles virus. It contained additional nucleotides “CGTACGATGACGTCCTAG” inserted just after nucleotide 3368 [the region between coding regions of P and Matrix protein (M) genes] to introduce unique restriction sites with a disrupted site for *Pfl*2311/*Bsi*WI. Restriction site for *Pfl*2311 was restored by introducing a single base substitution by site directed mutagenesis (SDM) and resulting virus generated from this genome was annotated as rMV.

### Site Directed Mutagenesis

Restriction site for *Pfl*2311 present in p(+)MV-NSE-FlagP-M502-3p construct was disrupted due to a single nucleotide substitution at position 3373 (g3373c). For restoration of restriction site, specific primers were designed to introduce the single point mutation by SDM. Primers were designed using online Quickchange primer design program (Agilent technologies, USA) with following 5′-3′ sequence [(c467g-FP:TTGTACTAGGACTCATCGTACGCTAGTTGGGTAT) and (c467g-RP:ATACCCAACTAGCGTACGATGACGTCCTAGTACAA)]. Mutagenesis was carried out using Herculase polymerase (Agilent technologies, USA) for amplification and *Dpn*I restriction enzyme (Thermofischer scientific, USA) for digestion of parental plasmid.

For generation of reporter virus, mCherry gene encoding for a red fluorescent protein was amplified from pmCherry-C1 plasmid (Clontech, USA) with specific primers (FP: 5′ATATCGTACGACCATGGTGAGCAAGGGCGAGG3′, RP: 5′TATAGACGTCTTACTTGTACAGCTCGTCCATG3′). Amplified product was inserted into full length measles viral genome between coding regions of phosphoprotein and matrix protein using *Pfl*2311 and *Aat*II restriction sites generating recombinant virus with reporter gene mCherry inserted and annotated as rMV-mCherry. Similarly, armed virus was generated by replacing the mCherry gene with BNiP3. Breast carcinoma cells were treated with apoptosis inducers to induce pro-apoptotic gene expression. Total RNA was isolated from drug treated cells, reverse transcribed and cDNA was amplified with BNiP3 specific primers: (FP:5′ATATCGTACGACCATGTCGCAGAACGGAGC3′, RP:5′TATAGACGTCTCAAAAGGTGCTGGTGG3′). Primarily, BNiP3 amplicon was purified and cloned into pcDNA3.1(+) expression vector between *Bam*HI and *Xho*I restriction sites. After confirmation of apoptotic effects in breast cancer cells following transfection with recombinant BNiP3 construct and infection with rMV (data not shown); BNiP3 gene was further sub-cloned into full length measles virus genome replacing mCherry gene to generate rMV-BNiP3 virus as described above.

### Rescue of rMV, rMV-mCherry, and rMV-BNiP3 Recombinant Viruses

The recombinant viruses were rescued using reverse genetics system established for measles virus by Radecke et al. (24). Briefly, HEK293 based packaging cell line stably expressing measles virus N, P, and T7 was generated. HEK293 cells were co-transfected with recombinant constructs of N, P, and T7 RNA polymerase using Lipofectamine 2000 (Life technologies, Invitrogen, USA). Cells were selected at a final concentration of 0.6 mg/ml G418 (Life technologies, Invitrogen, USA) and clones were screened for expression. Packaging cell line thus was co-transfected with recombinant measles virus L polymerase and measles virus full length viral genome [p(+)MV-NSE-FlagP-M502-3p] constructs. At 48 h post transfection, cells were harvested and recombinant virus particles were collected following three freeze-thaw cycles and stored at −80°C until further propagation. Recombinant measles virus harboring reporter gene (rMV-mCherry) or BNiP3 (rMV-BNiP3) were rescued similarly. Replication competency of recombinant virus particles rescued above was evaluated in breast cancer cells, MCF-7, and MDA-MB-231.

### Propagation and Production of rMV, rMV-mCherry, and rMV-BNiP3 Viruses

The lysates collected above were centrifuged at 1,200 g for 10 min at 4°C, filtered through a 0.22 micron filter and used as inoculum for infection. Around 0.1 × 10^6^ cells/ml MCF-7 or MDA-MB-231 and MCF-7 cells were seeded into a T25 cm^2^ flask and next day were infected with the filtered lysate. Infected cells were incubated at 37°C with 5% CO_2_ and observed for 4–5 days till the manifestation of morphological changes. Cells were then harvested and the replication competency of recombinant viruses generated was measured by checking the viral gene expression. However, reporter gene mCherry expression was directly visualized under fluorescent microscope.

RT-PCR: Total RNA was isolated from MCF-7 or MDA-MB-231 cells infected with recombinant measles viruses; rMV and rMV-BNiP3 independent of each other. Fraction of total RNA was reverse transcribed and cDNA was amplified with primers specific to genes encoding virus N, P, M, and BNiP3; foreign gene that was inserted into viral genome.

Immunoassays: Viral gene expression at protein level was checked by IFA staining and immunoblotting. MCF-7 and MDA-MB-231 cells grown on coverslips were used for IFA staining and cell lysates recovered from recombinant virus infected cells were subjected to SDS-PAGE followed by immunoblotting using anti-viral and anti-BNiP3 specific antibodies (mouse, Santacruz, USA). IFA slides were visualized under a Confocal Laser Scanning Microscope (Olympus FluoviewTM-FV1000) equipped with HeNe laser (488 nm) and pulsed diode laser (408 nm). Images were acquired with PLAPON 10X, 20X, 60X O NA: 1.42 oil immersion objective using FV10SW1.7 software.

Recombinant measles virus gene expression was checked for two to three consecutive rounds of infection in breast cancer cells to confirm the replication ability and progeny production of recombinant viruses generated. Recombinant virus was propagated and the titer was determined using standard Reed-Muench method (25). Recombinant virus infections were done at an MOI of 2 for rest of the experiments. We also measured the effects of recombinant measles viruses generated on breast cancer cells using various multiplicities of infection. MCF-7 and MDA-MB-231 cells were infected with armed and unarmed viruses independent of each other at different MOIs ranged from 0.5 to 6. Percentage of viability of infected cells was measured at 56 h post infection.

### Drugs Used

Synthesized hybrid hydrazone derivative, H2[N'-(2-chlorobenzylidene)-4- (2-(dimethylamino)ethoxy) benzohydrazide] having dimethylaminoethoxy tail structurally similar to Tamoxifen, a known anti-cancer chemotherapeutic, was a kind gift from Dr. Amir Azam from Department of Chemistry, Jamia Milia Islamia, New Delhi (26). Paclitaxel, a chemotherapeutic drug (Sigma-Aldrich) and H2 compound were reconstituted in 100% DMSO and further dilutions were made in serum free DMEM. To determine IC_50_ values of the drugs in, MDA-MB-231 and MCF-7 cells were grown up to 90% confluency, treated with increasing concentration of H2 from 0.5 to 200 μM and paclitaxel from 0.05 to 1 μM in triplicates. Medium devoid of drug was added to control wells. At 56 h post drug treatment, MTT assay was performed, and the values obtained were plotted in graph against the concentration of drug. Sub-lethal dose of both the drugs was chosen to study their combinatorial anti-cancer effects with rMV-BNiP3.

### *In vitro* Cell Viability Assay

MCF-7 and MDA-MB-231 cells were seeded in a 96-well plate at a density of ~10,000 and 20,000/well, respectively. Next day cells were infected with rMVor rMV-BNiP3 and incubated for 2–3 days. For viability assay of cells treated with combination of virus and drug, cells were first infected and then the drug was introduced after 2 h viral adsorption and post-adsorption cells were constantly exposed to the drug compounds. At 2–3 days post infection, infected cells were subjected to MTT assay, percentage of viability was calculated, and the graph was plotted.

### Detection of Proliferation Markers

MCF-7 and MDA-MB-231 cells were grown on coverslips and infected with rMV or rMV-BNiP3 followed by drug treatment as described above. At 48 h post infection, cells were fixed with chilled acetone, and subjected to IFA staining using anti-PCNA antibody (primary mouse, Santacruz, USA) for 2 h at 37°C and FITC-conjugated anti-mouse secondary antibody (goat, Sigma, USA) for 1 h at 37°C. Slides were visualized as mentioned earlier. Number of cells positive for nuclear antigen analyzed by Image J software was plotted with the mean values.

### Caspase 3 Activity Assay

MCF-7 and MDA-MB-231 cells were seeded at 0.2–0.25 × 10^5^ cells in 24-well plates. At 80% confluency, cells were infected with rMV or rMV-BNiP3. Two hours post viral adsorption, cells were washed once with serum free medium, and replenished with medium containing 2% FBS and desired concentration of paclitaxel or H2 compound. At 24 h post treatment, induction of apoptosis was measured using EnzCheck caspase 3 apoptosis kit (Life technologies, USA) as per manufacturer's instructions. Briefly, treated cells were harvested and lysed; 50 μl of lysate was incubated with specific substrate for 30 min at RT and the fluorescence was measured at 342/441 nm excitation-emission spectra with VarioskanFlash microplate reader (4.00.53) using SkanIt software 2.4.5 RE. Fluorescence detected was a direct measure of caspase 3 activity.

### Annexin V Staining

MDA-MB-231 and MCF-7 cells were seeded in 12-well plates at a density of 0.1 × 10^6^ cells/well. At ~80% confluency cells were infected with rMV or rMV-BNiP3 and incubated for 2 h for virus adsorption. Post-adsorption, desired concentration of paclitaxel (30 nM) or H2 (20 μM) compound was introduced into infected cells independent of each other. Infected cells were harvested at 24 h post drug treatment, washed with ice cold 1X PBS and processed for FACS analysis using Alexa fluor 488 Annexin V/Dead cell apoptosis kit (Invitrogen, USA). In brief, harvested cells were re-suspended in 100 μl of 1X annexin binding buffer; incubated with propidium iodide (PI) and Alexa fluor 488 conjugated annexin V for 15 min at RT. Volume was made up to 400 μl with 1X annexin binding buffer and cells were analyzed by FACS using BD AriaFusion with DiVa ver. 8.0.1(excitation with 488 nm laser and emission at 530 and 575 nm).

### Statistical Analysis

Analysis of the data was carried out by Graph Pad Prism software (version 5.04). Every experiment including MTT assay was done in biological triplicates. Data are represented as means and standard deviations. In MTT assay, means of percentage of cell viability and in caspase activity assay, means of fluorescence in each group, was compared with its corresponding control by student's *t*-test. For PCNA staining, number of cells positive for nuclear antigen in nine fields was counted and means was compared with their corresponding controls by student's *t*-test. For annexin V staining, means of ratio of annexin V/PI stained, and annexin V stained population was compared with their corresponding controls by student's *t*-test. A *p* < 0.05 was considered significant.

## Results

### Generation of Packaging Cell Line and Rescue of Recombinant Measles Virus

**Figure 1:**
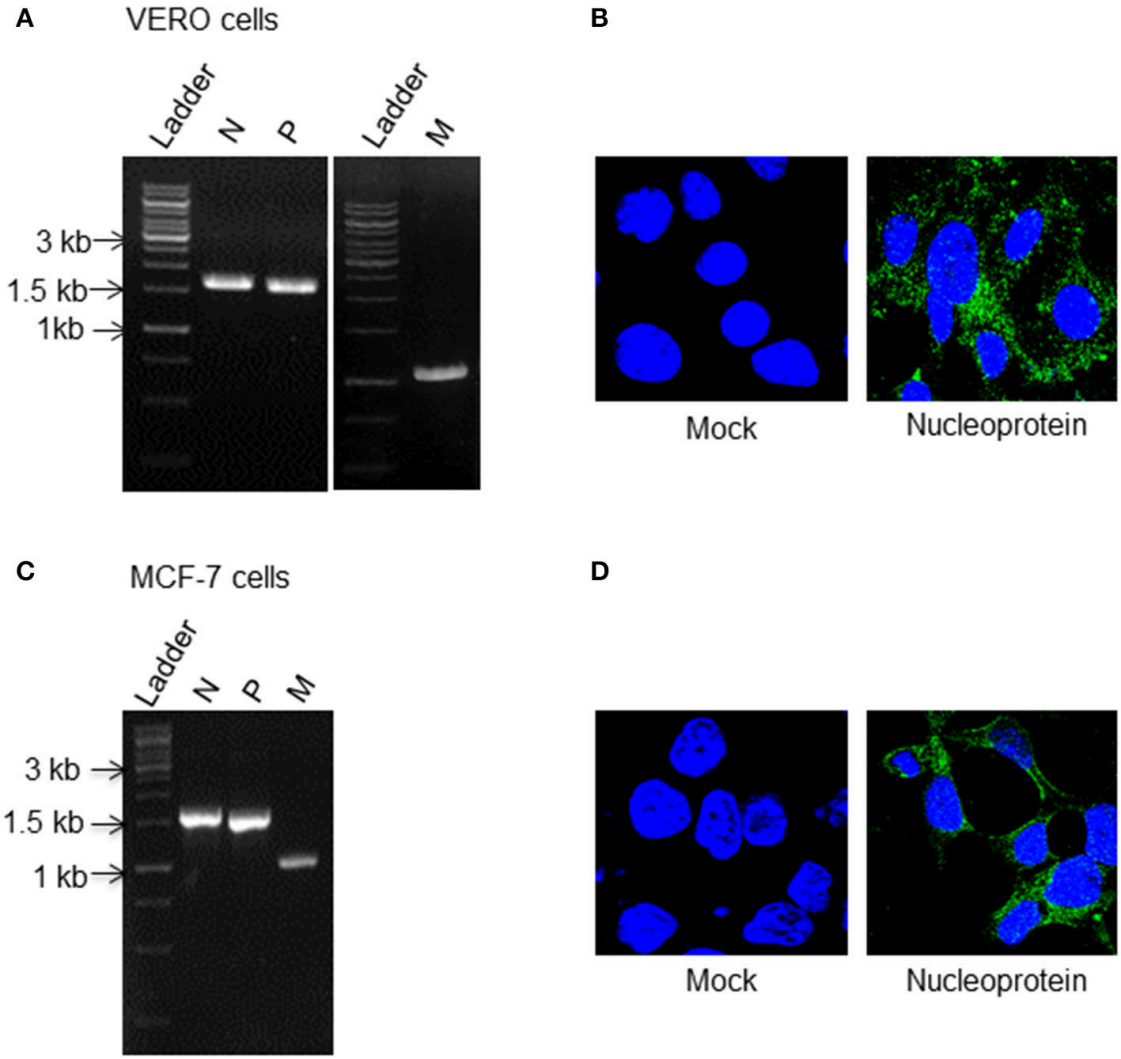
Replication competency of rescued recombinant measles virus (rMV). HEK293 cells stably expressing measles virus N, P, and T7 RNA polymerase were co-transfected with recombinant L plasmid (measles virus RNA dependent RNA polymerase) and measles virus full length genome, p(+)MV-NSE-FlagP-M502-3p (pMV) construct. Forty eight hours post transfection, cells were harvested and recombinant measles virus particles were rescued. To check the gene expression and the replication ability of recombinant virus recovered from packaging cell line; Vero and MCF-7 cells were infected with rescued virus and at 48 h post infection viral gene expression was checked. Viral gene expression in Vero and MCF-7 cells was checked for two successive passages and the images given here show the viral gene expression in the second passage. Expression of **(A)** N, P, and M transcripts by RT- PCR **(B)** N by IFA staining in Vero cells, **(C)** N, P, and M transcripts **(D)** N protein expression in MCF-7 cells.

In order to generate and rescue a recombinant measles virus, we followed the system developed by Radecke et al. (24) where an HEK293 based packaging cell line stably expressing measles virus N, P, and T7 was developed (Figure S1) and co-transfected with p(+)MV-NSE-FlagP-M502-3p obtained from Addgene and recombinant measles virus L (RNA dependent RNA polymerase) construct. To establish the successful reproduction of reverse genetics paradigm, Vero cells were infected with lysates and supernatants recovered from transfected cells. Expression of viral transcripts and proteins in Vero cells in subsequent passages confirmed the rescue of viable virus particles capable of replication (Figures 1A,B). Presence of early (N and P) and late (Matrix protein) viral gene transcripts was noted both 24 and 48 h post infection and showed increased levels with each passage. We also ascertained the infective potential of rescued recombinant virus (rMV) in breast cancer cells by infecting MCF-7 and observed for presence of viral transcripts and proteins (Figures 1C,D). Given images show the expression of viral genes in Vero and MCF-7 cells in the second passage.

### Generation of mCherry Reporter Virus and Armed Virus With Pro-apoptotic Gene

One of the major aims of this study was to generate an oncolytic virus armed to overcome the abrogated apoptotic machinery of cancer cells and to induce an onset of apoptosis leading to cancer cell death. Prior to insertion of BNiP3 into full length genome; reporter gene mCherry was inserted. This was done to test the stability of virion following insertion of a foreign gene and to evaluate its replication ability and viral gene expression through visualization of mCherry expression, a red fluorescent protein.

As stated earlier, recombinant full length measles virus genome has unique restriction site *Pfl*2311 between the region encoding P and M protein genes. As this site was observed to be disrupted, it was restored by inserting a point mutation at nucleotide position 3373 (c3373g) of p(+)MV-NSE-FlagP-M502-3p by SDM. The success of SDM was noted by the linearization of concerned vector upon digestion with *Pfl*2311 restriction enzyme. We used this restored site in combination with restriction site for *Aat*II to insert a gene encoding for either mCherry, a reporter gene or BNiP3 to generate recombinant virus particles; rMV-mCherry or rMV-BNiP3, respectively. Recombinant viral genome harboring mCherry or BNiP3 was confirmed by restriction enzyme digestion and sequencing (Figure S2).

Both the viruses were rescued as stated above and the generation and replication of rMV-mCherry virus was confirmed in cells through direct visualization of red fluorescence in Vero, MCF-7, and MDA-MB-231 cells (Figure S3). Expression of viral phosphoprotein in rMV-mCherry infected cells further confirmed the permissibility of armed virus with reporter gene (data not shown). However, successful generation of recombinant virus harboring BNiP3 was evaluated in MCF-7 and MDA-MB-231 cells. Expression of viral genes and foreign gene following infection of rMV-BNiP3 was confirmed in cells through detection of transcripts by RT-PCR and proteins by western blot analysis and IFA staining using specific antibodies to measles virus early protein, N; late protein, H, and BNiP3, thus establishing infective and replication competency of rMV-BNiP3 in breast cancer cells. MOI dependent cell death was noted in MCF-7 and MDA-MB-231 cells following infections with rMV or rMV-BNiP3 at different MOIs (Figure S4). During these preliminary experiments we found an indication of MDA-MB-231 cells being more sensitive to rMV-BNiP3, armed recombinant measles virus as compared to MCF-7 cells.

### Arming Does Not Affect the Replication Competency of rMV-mCherry and rMV-BNiP3

Numerous earlier studies have established that arming of measles virus neither negatively affect nor alter the replication competency of virus nor causes loss of its cytopathic effect on infected cells. We also observed similar results in MCF-7 and MDA-MB-231 cells infected with rMV-BNiP3. Armed virus showed enhanced cytopathic effect in MDA-MB-231 cells as compared to MCF-7 cells strengthening our earlier finding of the virus showing greater efficiency of either viral infection or replication in MDA-MB-231 cells. Results demonstrated the ability of virus to infect cancer cells which can be attributed to expression of CD46 and nectin-4 receptors in cancer cells. It may be noted that in addition to the role played by overexpression of the mentioned receptors in the establishment of infection, there could be certain internal factors enhancing the effects of virus in MDA-MB-231 cells which differ from MCF-7 cells.

**Figure 2:**
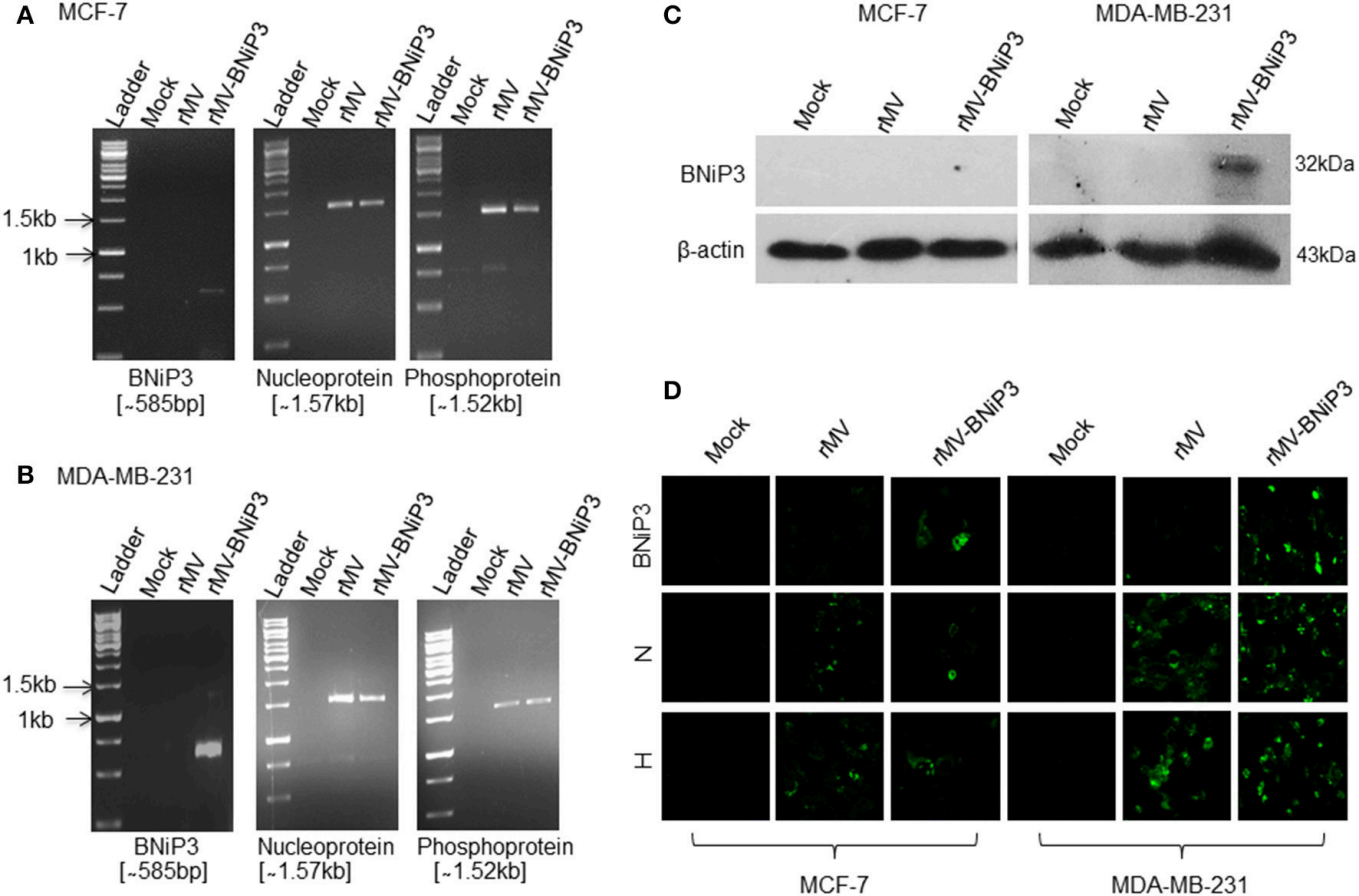
Confirmation of gene expression of rMV-BNiP3 virus following infection in breast cancer cells. MCF-7 and MDA-MB-231 cells were infected with rMV-BNiP3 and incubated for 48 h. Cells were then harvested, total RNA isolated, reverse transcribed, and cDNA was subjected to RT-PCR using specific primers to armed gene BNiP3 and viral genes N and P. Expression of BNiP3 (585bp), N (1.57kb), and P (1.52kb) was confirmed in **(A)** MCF-7 **(B)** MDA- MB-231 cells. To measure the viral and armed gene expression at protein level, MCF-7, and MDA-MB-231 cells were infected and at 48 h post infection cells were subjected to western blot analysis and IFA staining using anti-BNiP3, anti-viral N, and H antibodies. **(C)** Expression of BNiP3 in MCF-7 and MDA-MB-231 cells by western blot analysis, and **(D)** expression of BNiP3, N, and H by IFA staining.

As expected, increased length of the viral genome, more than its unit length, due to insertion of foreign gene (BNiP3) did not affect the infectivity and replication competency of rMV-BNiP3 in subsequent infections. However, in comparison to MCF-7 cells infected with rMV-BNiP3, infected MDA-MB-231 cells showed higher expression of BNiP3 as well as viral genes at transcript level (Figures 2A,B) and at protein level (Figures 2C,D). Higher expression levels of BNiP3 and viral genes including the hemagglutinin protein in MDA-MB-231 cells suggest higher affinity of rMV-BNiP3 toward TNBC cells.

### Combination of rMV-BNiP3 and Chemotherapeutic Agents Caused Heightened Toxicity in Breast Cancer Cells

Recent developments in the field of oncolytic virus medicine suggest a combinatorial role of viruses with an established anti-cancer regimen. To demonstrate the effectivity and applicability of rMV-BNiP3 in a combinatorial approach toward oncolysis, we had chosen paclitaxel, an established chemotherapeutic agent. In addition, we also explored the compatibility of a hybrid hydrazone derivative; H2 [N'-(2-chlorobenzylidene)-4-(2-(dimethylamino)ethoxy)] (26) with rMV-BNiP3 as an anti-tumor approach against breast carcinoma cells.

First, we calculated IC_50_ values for both paclitaxel and H2 against MCF-7 and MDA-MB-231 cells keeping HEK293 cells as normal cell control. As expected, paclitaxel being an already approved chemotherapeutic agent for treatment of various carcinomas including breast carcinoma showed higher toxicity at low concentrations in cancer cells as compared to normal cells. The IC_50_ values correspond to 99.48 nM in MCF-7 and 88.89 nM in MDA-MB-231 cells. In comparison, H2 compound showed anti-tumor toxicity at a much higher IC_50_ value of 80 μM in MCF-7 and 90 μM in MDA-MB-231 cells. Interestingly, H2 showed very low level of toxicity in HEK293 cells, where we recorded IC_50_ at a very high concentration of 230 μM. In all our further experiments combination of recombinant virus either with paclitaxel or H2 compound was used. We used a sub-lethal dose of 30 nM paclitaxel and 20 μM H2.

**Figure 3:**
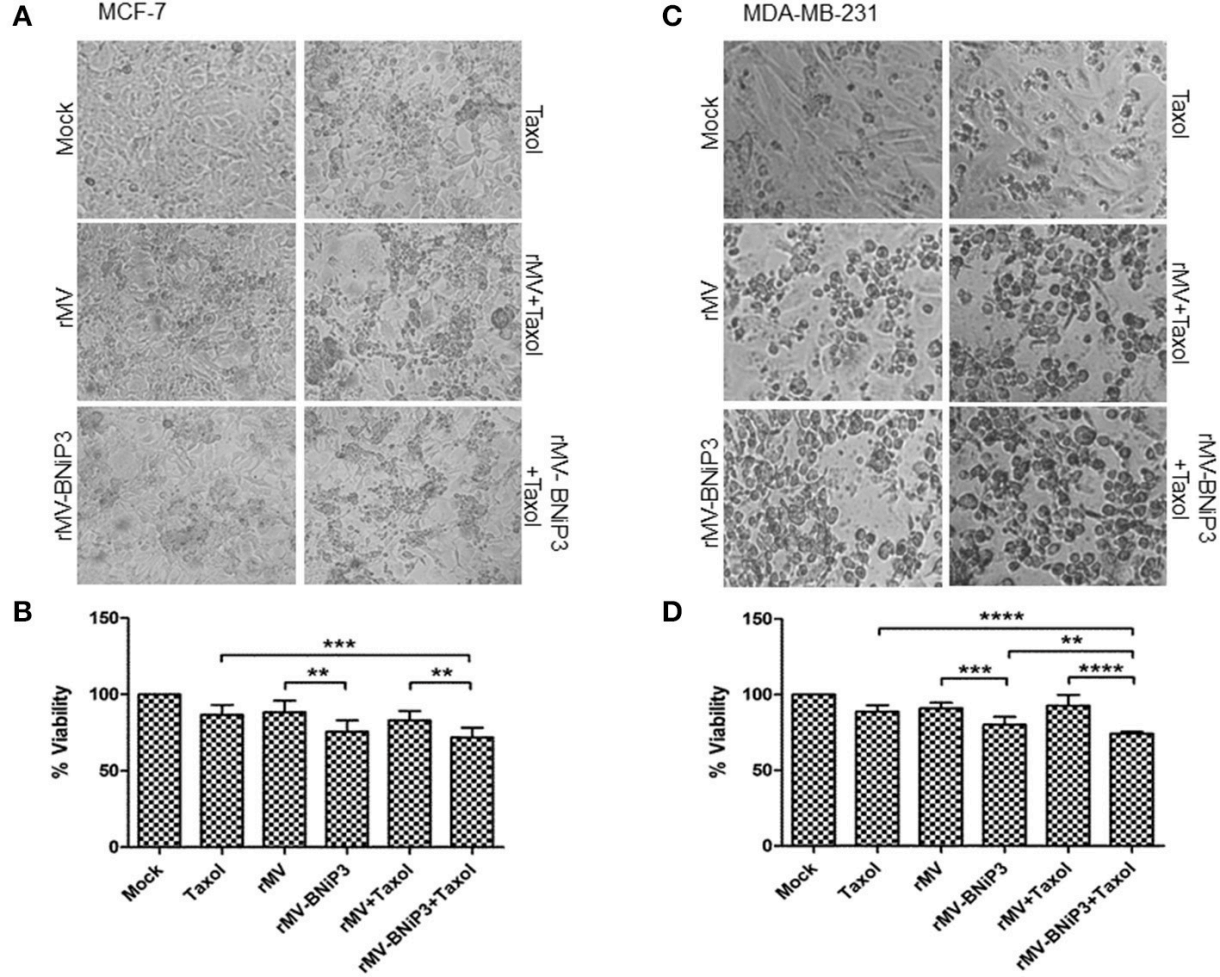
Combined effect of paclitaxel and recombinant virus in breast cancer cells. MCF-7 and MDA-MB-231 cells were infected with rMV or rMV-BNiP3 in the presence of desired concentrations of paclitaxel. Cells infected either with rMV or rMV-BNiP3 or treated with drug alone were used as control for comparison. Infected cells were incubated at 37°C; CO_2_ atmosphere and images of morphological changes induced were taken with microscope and compared with the respective controls. At 56 h post infection, cells were subjected to MTT assay to measure the percentage of cell viability. **(A,C)** Photomicrographs showing morphological changes induced following infection and drug treatment in MCF-7 and MDA-MB-231 cells, respectively. **(B,D)** Percentage of viability of infected MCF-7 and MDA-MB-231 cells treated with paclitaxel, respectively. ^**^p ≤ 0.01; ^***^p ≤ 0.001 and ^****^p ≤ 0.0001.

**Figure 4:**
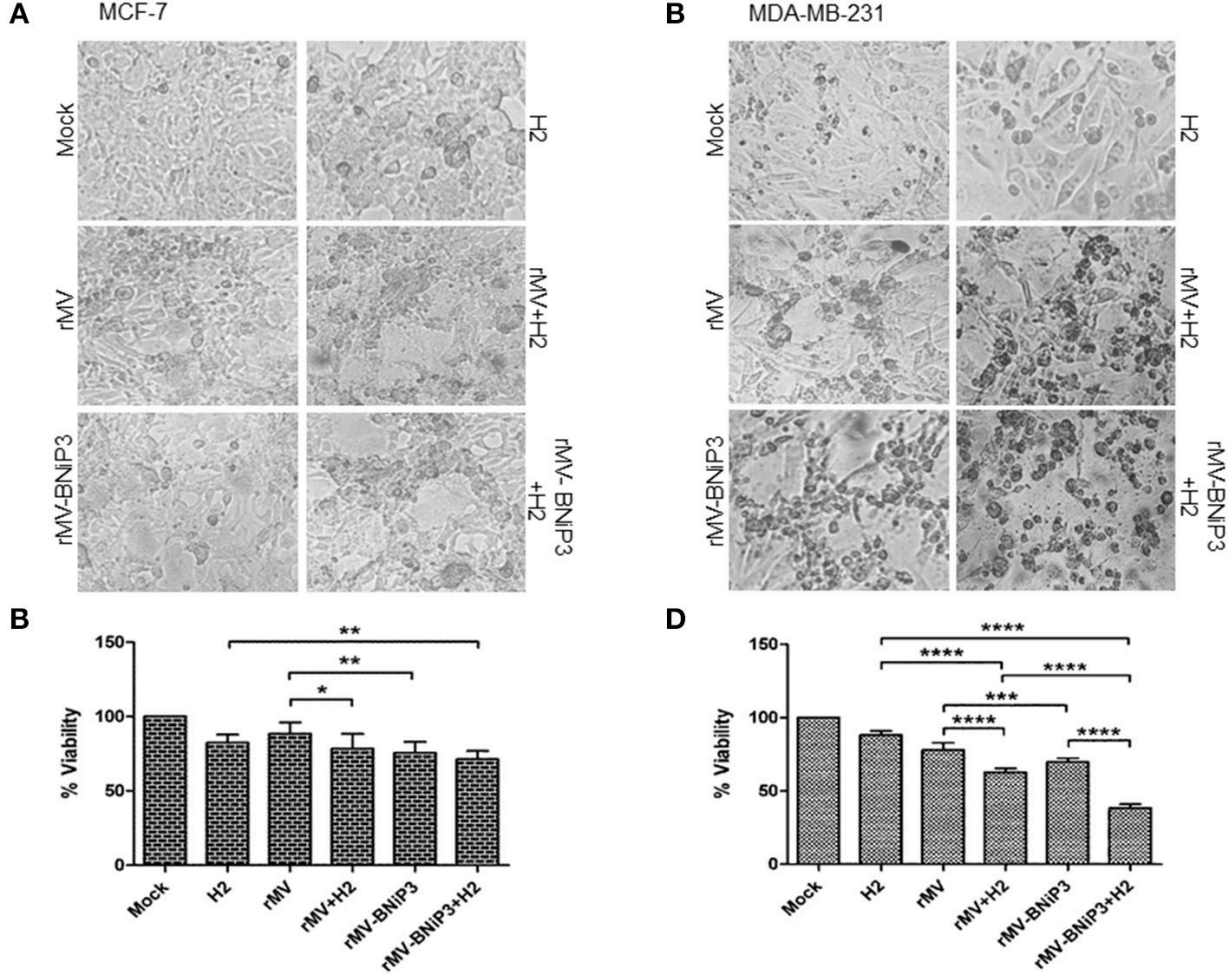
Combined effects of recombinant virus and H2 compound in breast cancer cells. MCF-7 and MDA-MB-231 cells were infected with rMV-BNiP3 followed by H2 compound treatment. Cells infected either with rMV or rMV-BNiP3 or treated with drug alone were used as controls. Infected cells were incubated at 37°C; CO_2_ atmosphere and images of morphological changes induced were taken with microscope and compared with the respective controls. At 56 h post infection, cells were subjected to MTT assay to measure the percentage of cell viability. **(A,C)** Photomicrographs showing morphological changes induced following infection and drug treatment in MCF-7 and MDA-MB-231 cells, respectively. **(B,D)** Percentage of viability of infected MCF-7 and MDA-MB-231 cells treated with H2, respectively. ^*^p ≤ 0.05; ^**^p ≤ 0.01; ^***^p ≤ 0.001 and ^****^p ≤ 0.0001.

As a preliminary measure of whether rMV-BNiP3 has any effect on cell death and survival of breast cancer cells, we performed MTT based cytotoxicity assay on both MDA-MB-231 and MCF-7 cell lines infected with rMV-BNiP3. Both the cell lines were infected with rMV-BNiP3 in the presence of either paclitaxel or H2 compound. Cytotoxicity was measured 56 h post-infection. Keeping in accord with our previous observations, morphological changes such as shrinkage and rounding of adherent cells were observed to be high in paclitaxel treated rMV-BNiP3 infected cells as compared to their respective controls (Figures 3A,C). Corresponding to the observed morphological changes, the measured toxicity of rMV-BNiP3 infected MDA-MB-231 and MCF-7 cells treated with paclitaxel was significantly higher as compared to cells either infected with rMV-BNiP3 (*p* = 0.0093) or cells treated with paclitaxel (*p* ≤ 0.0001) alone (Figures 3B,D). Independent of paclitaxel treatment, significant toxicity was recorded in MDA-MB-231 (*p* = 0.0003) and MCF-7 cell lines (*p* = 0.0045) following rMV-BNiP3 infection as compared to rMV infection. It suggests that the armed virus has relatively higher oncolytic activity than unarmed virus. MCF-7 cells infected with rMV-BNiP3 had demonstrated similar effects following combined treatment with paclitaxel (*p*=0.0004) as compared to cells treated with drug alone. Morphological changes induced in response to rMV-BNiP3 infection combined with H2 compound were observed to be similar to paclitaxel treatment in MCF-7 and MDA-MB-231 cells (Figures 4A,C). The measured toxicity of MDA-MB-231(*p* ≤ 0.0001) and MCF-7 (*p* ≤ 0.0013) cells was significantly increased in cells treated with combination of rMV-BNiP3 and H2 compound as compared to H2 treatment alone (Figures 4B,D). *P*-values corresponding to significance of each experiment between two parameters is shown in Table 1.

**Table 1:** Significant values corresponding to cell toxicity assay.

**Sample population**	**MDA-MB-231 *p*-value**	**MCF-7 *p*-value**
rMV vs. rMV-BNiP3	0.0003 (S)	0.0045 (S)
rMV vs. rMV+Taxol	0.5706 (NS)	0.1434 (NS)
rMV-BNiP3 vs. rMV-BNiP3+Taxol	0.0093 (S)	0.2876 (NS)
Taxol vs. rMV+Taxol	0.1999 (NS)	0.2711 (NS)
Taxol vs. rMV-BNiP3+Taxol	< 0.0001 (S)	0.0004 (S)
rMV+Taxol vs. rMV-BNiP3+Taxol	< 0.0001 (S)	0.0033 (S)
rMV vs. rMV+H2	< 0.0001 (S)	0.0419 (S)
rMV-BNiP3 vs. rMV-BNiP3+H2	< 0.0001 (S)	0.2038 (NS)
H2 vs. rMV+H2	< 0.0001 (S)	0.3387 (NS)
H2 vs. rMV-BNiP3+H2	< 0.0001 (S)	0.0013 (S)
rMV+H2 vs. rMV-BNiP3+H2	< 0.0001 (S)	0.1030 (NS)

Irrespective of drug treatment, both MCF-7 and MDA-MB-231 cells were observed to be sensitive to rMV-BNiP3 virus infection than unarmed virus. However, the oncolytic effects exerted to above combination in triple negative breast cancer cells, MDA-MB-231 was recorded to be relatively higher than MCF-7 cells. This can be attributed to the higher sensitivity of MDA-MB-231 cells toward rMV-BNiP3 as compared to MCF-7 cells.

**Figure 5:**
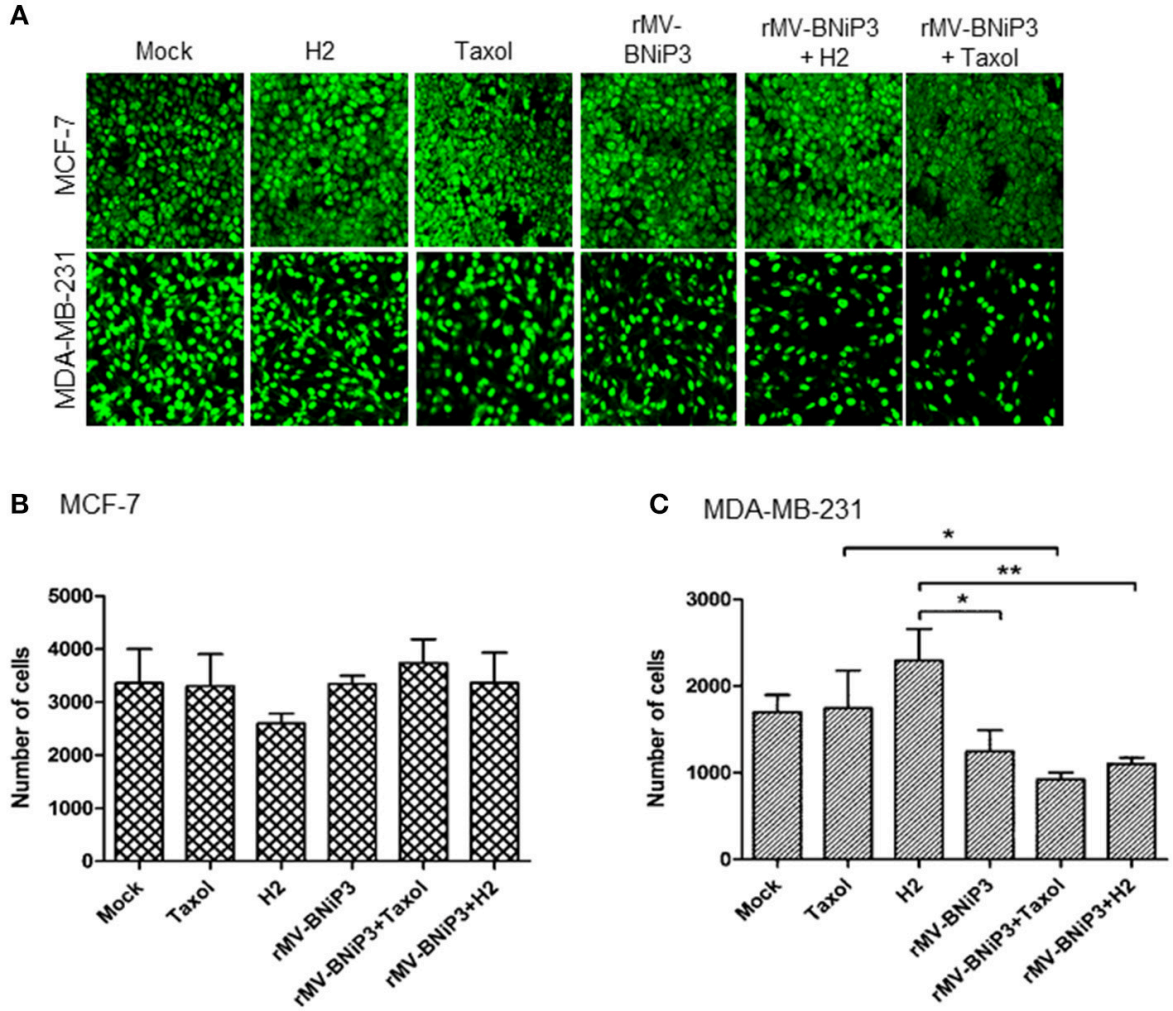
Expression of PCNA as measurement of proliferative index. MCF-7 and MDA-MB-231 cells were grown on coverslips. At 80% confluency cells were infected with rMV-BNiP3 and treated with desired concentrations of paclitaxel or H2 compound. Cells were incubated at 37°C; 48 h post infection, cells were subjected to IFA staining with specific antibody to proliferative marker PCNA (proliferating cellular nuclear antigen). Nine different fields in each sample were analyzed by Image J software and the results were plotted in column graph with the mean values. **(A)** Expression of PCNA in MCF-7 and MDA-MB-231 cells, **(B,C)** Column graphs showing number of cells positive for nuclear antigen in MCF-7 and MDA-MB-231 cells, respectively. PCNA expression was significantly reduced in rMV-BNiP3 infected MDA-MB-231 cells treated with paclitaxel (*p* = 0.0326) or H2 (*p* = 0.051) as compared to cells treated with drugs alone. No significant effects were noted in MCF-7 cells. ^*^p ≤ 0.05 and ^**^p ≤ 0.01.

Results were further validated in infected cells by measuring the proliferative marker PCNA (proliferating cell nuclear antigen), an indicator of dividing cells. PCNA is a well-conserved protein expressed during DNA synthesis in cells that undergo division. Combined effects of virus and drug in MCF-7 and MDA-MB-231 cells were validated by IFA staining using antibodies specific to PCNA (Figure 5A). The number of PCNA stained nuclei was significantly reduced in MDA-MB-231 cells infected with rMV-BNiP3 in combination with paclitaxel (*p* = 0.0326) or H2 compound (*p* = 0.0051) as compared to their respective controls (Figures 5A,C). PCNA staining demonstrated decreased proliferation of MDA-MB-231 cells following combined application of drug and virus. However, no such significant difference was observed in MCF-7 cells (Figures 5A,B).

### Comparable Increase in Caspase 3 Activity Was Observed in Treated Breast Cancer Cells

**Figure 6:**
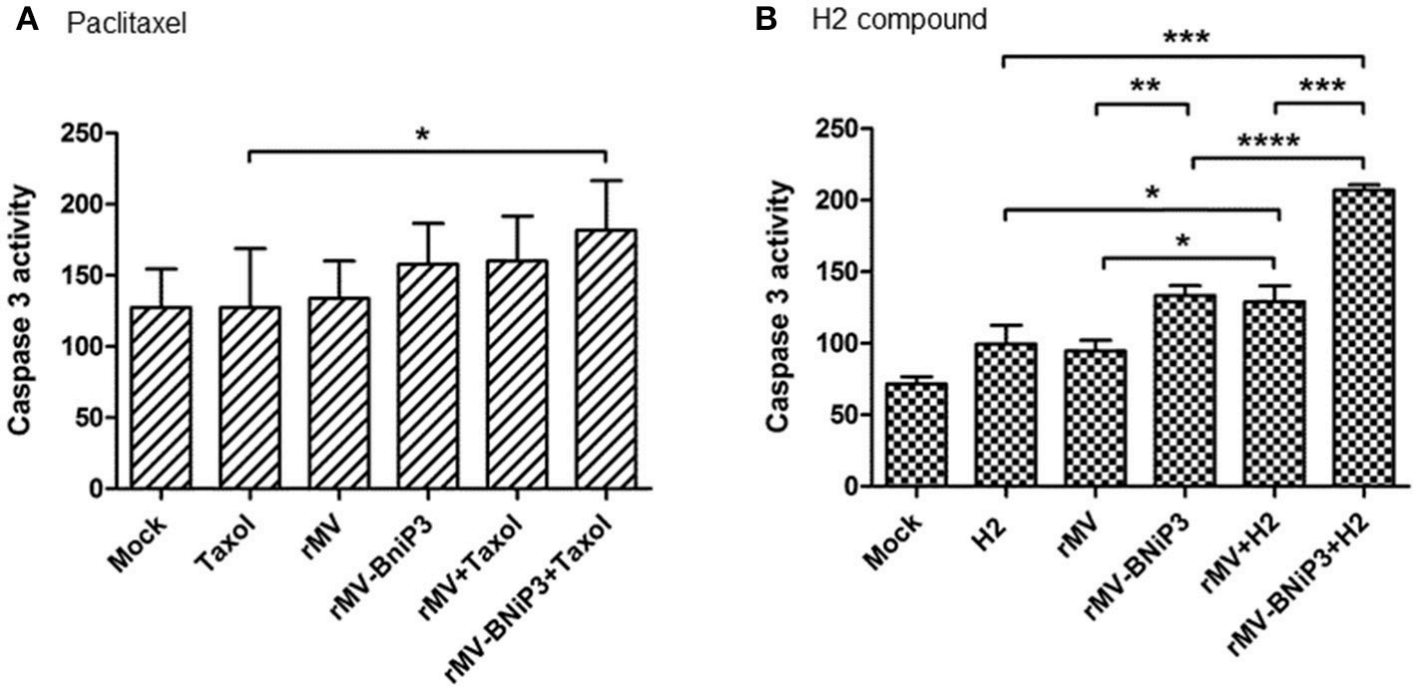
Caspase 3 activity in MDA-MB-231 cells infected with recombinant virus in combination with drugs. MDA-MB-231 cells were infected with rMV-BNiP3 followed by treatment with desired concentrations of paclitaxel or H2 compound. At 48 h post infection, caspase 3 activity, an indicator of apoptosis was measured. Caspase activity of TNBC cells treated with **(A)** paclitaxel and **(B)** H2 compound in combination with virus. Cells infected with rMV (*p* = 0.0395) and rMV-BNiP3 (*p* = 0.0002) in the presence of H2 compound showed significantly increased caspase 3 activity as compared to cells treated with H2 alone. ^*^p ≤ 0.05; ^**^p ≤ 0.01; ^***^p ≤ 0.001 and ^****^p ≤ 0.0001.

Caspase 3 activity was measured as a representation of caspase-dependent activation of apoptotic machinery in MDA-MB-231 cells infected with recombinant virus followed by paclitaxel or H2 compound treatment keeping mock infected cells as control. The oncolytic potential of rMV-BNiP3 was more pronounced in triple negative breast cancer cells as standalone and also in combination with drugs. It was observed that MDA-MB-231 cells treated with combination of rMV-BNiP3 and paclitaxel showed higher caspase 3 activity than cells treated with paclitaxel (*p* = 0.0130) alone (Figure 6A). In comparison, MDA-MB-231 cells treated with combination of rMV-BNiP3 and H2 also exhibited higher caspase 3 activity as compared to either rMV-BNiP3 (*p* ≤ 0.0001) or H2 (*p* = 0.0002) alone (Figure 6B). Similar to our earlier observations, rMV-BNiP3 infected MDA-MB-231 cells also had shown higher caspase 3 activity as compared to rMV infected cells (*p* = 0.0026) both in the case of standalone virus infections and combined treatment with H2 (*p* = 0.0003). These results correlated with our earlier observation of differential viral gene expression in both the cell lines and confirmed the better sensitivity of TNBC toward rMV-BNiP3.

### Induction of Apoptosis in Majority of Breast Cancer Cells Infected With rMV-BNiP3

**Figure 7:**
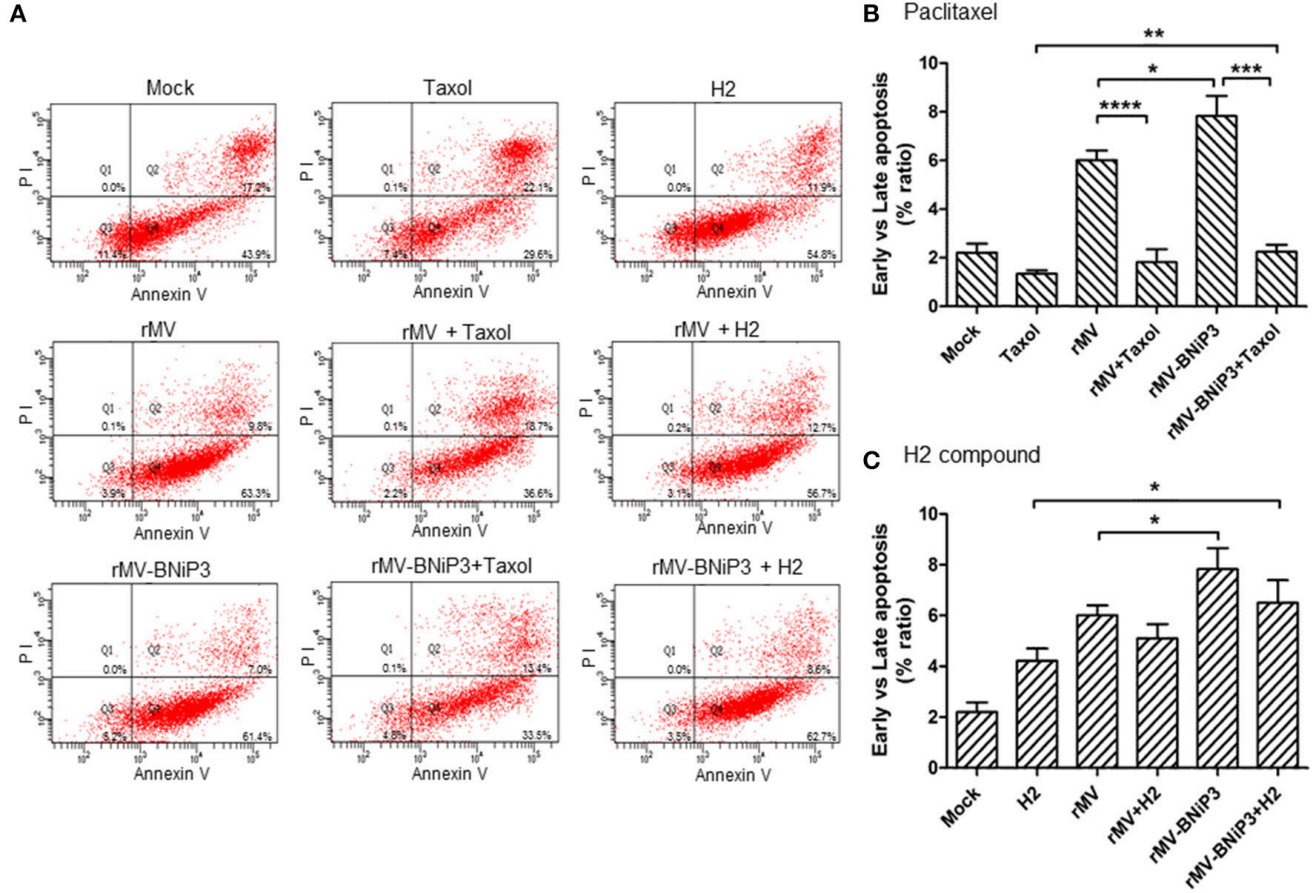
Annexin V staining of MDA-MB-231 cells infected with recombinant virus and treated with drugs. MDA-MB-231 cells were infected with rMV or rMV-BNiP3 then treated with desired concentration of paclitaxel and H2 compound. Infected cells were harvested at 24 h post drug treatment and processed for FACS analysis using Alexa fluor 488 Annexin V/Dead cell apoptosis kit. A total number of 10,000 were analyzed and the data represented as column graph indicates percentage ratio of Annexin V/PI staining vs. Annexin V stainingin rMV-BNiP3 infected cells as compared to rMV infected cells or cells treated with drug alone. **(A)** Quadrants representing annexin V and annexin V/PI stained cells. Percentage of annexin V positive cells treated with **(B)** paclitaxel **(C)** H2 compound. Population of annexin V positive cells were higher in cells infected with rMV-BNiP3 (*p* = 0.0261) as compared to rMV. Cells treated with combination of rMV-BNiP3 with H2 (*p* = 0.0175) showed higher population of annexin V positive cells as compared to H2 alone. Cells infected with rMV-BNiP3 followed by treatment with paclitaxel showed higher population of annexin V positive cells as compared to its drug control (*p* = 0.0077) but showed significantly lower population of annexin V positive cells (*p* = 0.0004) as compared to the respective virus controls due to higher population of PI positive dead cells. ^*^p ≤ 0.05; ^**^p ≤ 0.01; ^***^p ≤ 0.001 and ^****^p ≤ 0.0001.

As annexin V and PI are used as markers for apoptosis and cell death, respectively, we analysed MCF-7 and MDA-MB-231 cells infected with rMV-BNiP3 supplemented with paclitaxel (30 nM) or H2 (20 μM) prior to infection to ascertain rMV-BNiP3 induced apoptosis using FACS based Annexin V assay. The means of the percentage ratio of Annexin V and PI positive population (late apoptosis/dead cells) and Annexin V positive and PI negative (early apoptosis/viable cells) population was plotted for MDA-MB-231(Figures 7A–C) and MCF-7 cells (Figure S5). The percentage of Annexin V and PI positive cells was also plotted for MDA-MB-231 (Figure S6) and MCF-7 cells (Figure S7). The data represented as column graph indicates the increased level of apoptosis in rMV-BNiP3 (*p* = 0.026) infected cells as compared to rMV infected cells. Infection with rMV-BNiP3 in combination with paclitaxel had shown significantly lesser annexin V staining as compared to rMV-BNiP3 (*p* = 0.0004) alone (Figure 7B). This is due to a major population of cells undergoing death as evident from the higher amount of Annexin V and PI stained cells as compared to annexin V stained cells (Figure 7A and Figure S6). However, a significant increase in apoptosis was noted in cells treated with rMV-BNiP3 and paclitaxel (*p*=0.0077) as compared to paclitaxel alone. This difference could be due to relatively low apoptosis in paclitaxel treated cells over cells treated with the combination.

Likewise, following H2 treatment, rMV-BNiP3 infected cells had shown higher annexin V staining (*p* = 0.0175) as compared to cells treated with H2 only (Figure 7C). Increased apoptotic activity in rMV-BNiP3 infected cells suggests that the expression of BNiP3 in infected cells could contribute to anti-tumor activity in association with recombinant measles virus. Correlating with caspase 3 activity results, both paclitaxel and H2 compound did not induce any significant apoptotic effect as standalone treatment. However, in combination of BNiP3 armed virus, both the drugs induced significantly increased cell death and apoptosis respectively. In comparison to MDA-MB-231 cells, results obtained withAnnexin V staining of MCF-7 cells observed to be inconsistent (Figures S5, S7). Also, cytotoxicity recorded with MCF-7 cells following recombinant virus infection was not comparable with PCNA staining, caspase 3 activity, and Annexin V staining.

The mechanism of rMV-BNiP3 mediated cell death in triple negative cell line remains elusive, so our future studies will be focused on this aspect. But the scope of the current study was to construct a stable and replication competent recombinant virus armed with a foreign genetic element to enhance its oncolytic potential.

## Discussion

In the current study, we developed a measles virus based oncolytic agent to target the deregulated apoptotic pathway in cancer cells. It was done by inserting BNiP3, a pro-apoptotic gene, in measles viral genome to generate an armed oncolytic virus. We also demonstrated that this armed virus can be used in combination with paclitaxel, a widely used chemotherapeutic agent for various cancers including breast cancer, or H2, a hydrazone derivative which was observed to be selective to cancer cells over normal cell in our earlier studies (26), for effective oncolysis. Our study shows that cells treated with armed virus, rMV-BNiP3, elicited greater degree of apoptosis, and cell death as compared to cells infected with unarmed measles virus, rMV or uninfected cells. We hypothesize that the infection of rMV-BNiP3 in cancer cells will lead to expression of pro-apoptotic gene and the inability of cancer cells to suppress this expression will induce apoptosis. An interesting observation was the relatively higher activity of virus in MDA-MB-231 cells, a triple negative breast carcinoma cell line, as compared to ER+ MCF-7 cells.

Measles virus as an oncolytic agent is one of the fast developing therapy with some platforms already under phase II clinical trials. Examples can be sought in studies involving measles virus expressing NIS (sodium iodide symporter) which in combination with radioisotope of iodine (^131^I), sequesters it in the tumor and leads to lysis of radiosensitive cancer cells as seen in case of preclinical models of multiple myeloma (27), prostate cancer, glioblastoma multiforme, and medulloblastoma (28). Measles virus has also been armed with genes encoding for pro-drug activation such as purine nucleoside phosphorylase (PNP) from *E. coli* (29), with GMCSF and IFN β (30) and with IL-12 which acts as an immunomodulator and mediates anti-tumor effects through T-cell activation (31). Neutrophil activating protein of *Helicobacter pylori* inserted into viral genome acts as an immunomodulator to enhance the oncolytic activity of the virus (32). In these cases arming facilitated the efficient killing of metastatic cancer cells through cell to cell virus spread. All the above reports collectively suggest that the oncolytic potential of measles virus can be enhanced by insertional modifications.

Present study also establishes an “armed” oncolytic measles virus harboring BNiP3 gene between the coding region for measles virus P and M. Expression of BNiP3 at transcript and protein level in cells infected with rMV-BNiP3 confirmed the successful generation of a recombinant virus with extra genome than its unit length. Increased genome length neither affected its replication efficiency nor assembly and packaging. Of both the breast cancer cell line models used, more robust viral response was recorded in majority of assays done to establish the lytic activity of recombinant virus in MDA-MB-231 cells as compared to MCF-7 cells. The response of recombinant virus infection in MCF-7 cells was not consistent and variations were observed in replicate experiments as well.

Inclusion of BNiP3 as arming component of oncolytic measles virus came with the purpose to induce the ablated apoptosis machinery of cancer cells. BNiP3 is a Bcl-2/adenovirus E1B 19kDa interacting protein with a BH3 domain. It is a pro-apoptotic protein localized on the outer mitochondrial membrane, induced under hypoxia and is absent or downregulated in many cancers. BNiP3 functionality has been associated with caspase independent cell death without induction of cytochrome c release (33, 34) and induction of autophagy and mitophagy (35, 36). But it also plays a controversial role in tumor-progression in a cancer-type dependant manner. There have been examples of its role both as an anti and pro-tumorigenic protein. In some cases of breast carcinoma (5) and prostate cancer (37), elevated levels of BNiP3 were associated with increased metastasis and poor prognosis. Whereas, studies have also been shown increased metastasis and suppression of apoptosis attributed to suspension of mitophagy due to downregulation of BNiP3. Examples can be sought in case of pancreatic cancer (17) and some mammary tumors (38) where loss of BNiP3 function leads to increased metastasis. In case of TNBC, loss of BNiP3 function in context of elevated levels of HIFα cause poor prognosis for metastasis-free survival (18). It shows that BNiP3 dependant mitophagy plays an important role in induction of apoptosis in response to hypoxic and nutrient deprived conditions present in many tumors. These observations are more relevant in case of BNiP3 null tumors, which employ suppression of BNiP3 for their progression. Measles being a virus with its infective life cycle carried out specifically in cellular cytoplasm, showed BNiP3 expression in cytoplasm of infected MDA-MB-231 cells. This cytoplasmic expression has clinical efficacy due to the fact that there have been clinical reports linking downregulation and nuclear expression of BNiP3 to increased potential of metastasis in breast cancer patients (39).

Cytotoxicity experiments pertaining to activity of rMV-BNiP3 suggests its better oncolytic potential than rMV in both MCF-7 and MDA-MB-231 cells. The recorded cell death was significantly higher with rMV-BNiP3 as compared to rMV and was relatively higher in MDA-MB-231 cells as compared to MCF-7 cells. In our study, preliminary experiments performed in measuring apoptosis following transfection of BNiP3 construct and rMV infection had demonstrated increased caspase activity in both the cell lines (data not shown). But assays done to observe the induction of apoptosis in cells infected with the recombinant virus carrying BNiP3 gene had shown higher toxicity and caspase 3 activity than rMV in MDA-MB-231 cells. This observation was made more interesting with the fact that MDA-MB-231 cells are the triple negative population of breast cancer cells which neither expresses estrogen and progesterone receptors nor it is Her2/neu positive. Both the cell lines do express CD46 receptor but the infection ability and the relatively higher expression of viral genes at transcript and protein level including BNiP3 could be due to various cellular factors involved in the progression of viral infection providing favorable environment and better sensitivity to MDA-MB-231 cells toward rMV-BNiP3 as compared to MCF-7 cells. Tumor targeting is a major feature of the oncolytic virotherapy but viral tropism can be modified for efficient targeting of the oncolytic virus to tumor cells. No modification with respect to targeting was done to the recombinant virus generated in this study. However, the relatively higher activity of this virus in MDA-MB-231 cells and the host factors contributing to the better selectivity of rMV-BNiP3 to triple negative breast cancer cells are important features to be explored. The main aim of this study was to generate measles virus armed with a foreign gene and to check its effects both as a standalone and combination treatment module. Here, we have demonstrated arming of measles virus with BNiP3 to antagonize anti-apoptotic nature of breast tumor cells, enhances its lytic ability as compared to unarmed virus suggesting that BNiP3 contributes to increased apoptosis. Further studies to elucidate the mechanism of action of rMV-BNiP3 toward MCF-7 and MDA-MB-231 cells as well as signal pathways and their downstream molecules involved in its oncolytic activity would provide insight into the cause of its heightened response against triple negative breast cancer cells.

Oncolytic virus, as a monotherapy, still has ways to go before achieving greater success. Keeping this in mind we combined rMV and rMV-BNiP3 with a known drug and a hybrid hydrazone derivative. Inclusion of H2 compound, a hydrazone derivative was based on our previous work (unpublished data) where we had observed H2 selectivity to cancer cells. Generally paclitaxel is given as chemotherapy in various cancers, but as with all chemotherapeutic agents, it also has systemic side-effects. By treating the breast carcinoma cells with paclitaxel or H2 compound at a sub-lethal dose in combination with rMV or rMV-BNiP3, we observed increased caspase 3 activity. This increase was observed significantly in combinatorial treatment of virus and drug as compared to only virus, thus indicating synergism between the two modalities. In addition to caspase 3 activity, a significant decrease in PCNA, a nuclear proliferation marker, was also observed in MDA-MB-231 cells treated with a combination of rMV-BNiP3 and paclitaxel. This observation of synergism was made even more interesting when infection combined with a novel hydrazone derivative [N'-(2-chlorobenzylidene)-4-(2-(dimethylalamino)ethoxy) benzohydrazide)] H2, the cytotoxic effect against MDA-MB-231 increased to a significant level as compared to only rMV or rMV-BNiP3 infected cells. It may be noted that the working concentration of H2 used (20 μM) may seem quite improbable for actual use but its relatively low toxicity at even higher concentrations of ~240 μM (IC_50_ value) in HEK293 cells makes the utilized concentration of drug sub-lethal. Higher population of annexin V stained cells corresponding to apoptosis in rMV-BNiP3 infected MDA-MB-231 cells devoid of paclitaxel indicates the ability of rMV-BNiP3 to overcome the downregulated apoptotic machinery to induce apoptosis in infected cells. However, treatment of cells with paclitaxel didn't garner significant population of annexin V positive cells in rMV-BNiP3 or rMV infected cells as compared to their viral controls. It could be attributed to the fact that the cells treated with combination of virus and paclitaxel led to higher population of PI positive dead cells. This indicates enhanced oncolytic profile of combination therapy as compared to monotherapy with either drug or virus. Use of sub-lethal doses of paclitaxel or H2 and their contribution in death of cancer cells in combination with rMV-BNiP3 is indicative of the potential this therapy can have toward reducing the side effects associated with chemotherapeutic drugs. H2 being a relatively new compound, its activity and effectivity against cancer remains to be explored with *in vivo* settings. Our study is the first to explore the combination of oncolytic measles virus with paclitaxel.

In addition to the replication and propagation, the mechanism of action of an oncolytic virus is a complex process. It does not solely depend on virus-induced lysis after host machinery exhaustion to induce tumor-cell death but also incorporates the host immune response for effective oncolysis. Studies have demonstrated the major role immunogenic cell death plays in oncolytic virus induced cancer cell death (40). In case of rMV-BNiP3, lack of immune component, and responsiveness of an *in vitro* cellular platform may have played a role in the comparable anti-cancer effect observed with rMV and rMV-BNiP3. Thus, it remains to be seen that whether it has an effective oncolytic role in case of *in vivo* tumor models or whether it potentiates the apoptotic response toward invasion.

## Conclusion

This study may have practical implications for the fact that it provides a model for effective treatment of TNBC, a subtype of breast carcinoma often presenting with metastasis with limited responsiveness toward available therapeutics. Since our study shows increase in toxicity and apoptotic activity of cells treated with rMV-BNiP3 as a standalone or in combination with sub-lethal doses of paclitaxel and H2, it can emerge as a therapeutic tool with reduced drug-associated side effects.

## Author Contributions

MR designed the study and supervised the work. GL performed the experiments. MR and GL analyzed the data, prepared the manuscript, and approved the manuscript.

### Conflict of Interest Statement

The authors declare that the research was conducted in the absence of any commercial or financial relationships that could be construed as a potential conflict of interest.

## Abbreviations

TNBC: Triple negative breast carcinoma
rMV: recombinant measles virus
rMV-mCherry: recombinant measles virus harbouring mCherry reporter gene
rMV-BNiP3: recombinant measles virus harboring BNiP3 gene
Taxol: Paclitaxel
H2, N'-(2-chlorobenzylidene)-4-(2-(dimethylalamino)ethoxy) benzohydrazide), N: Nucleoprotein
P: Phosphoprotein
T7: T7 RNA Polymerase
M: Matrix protein
H: Hemagglutinin
PI: Propidium iodide.
