# Supplementary material for: Combination of Oncolytic Measles Virus Armed With BNiP3, a Pro-apoptotic Gene and Paclitaxel Induces Breast Cancer Cell Death
Source: Front Oncol. 2019 Jan 15;8:676. doi: 10.3389/fonc.2018.00676 (PMC6340943; doi:10.3389/fonc.2018.00676)
Supplement: Supplementary file 1 [file Data_Sheet_1.pdf]

Supplementary Material

Combination of oncolytic measles virus armed with BNiP3, a pro-apoptotic gene and paclitaxel induces breast cancer cell death

Geetanjali Lal, Maitreyi S. Rajala*

*** Correspondence:** Dr. Maitreyi S. Rajala: msrajala@mail.jnu.ac.in

Supplementary Figures

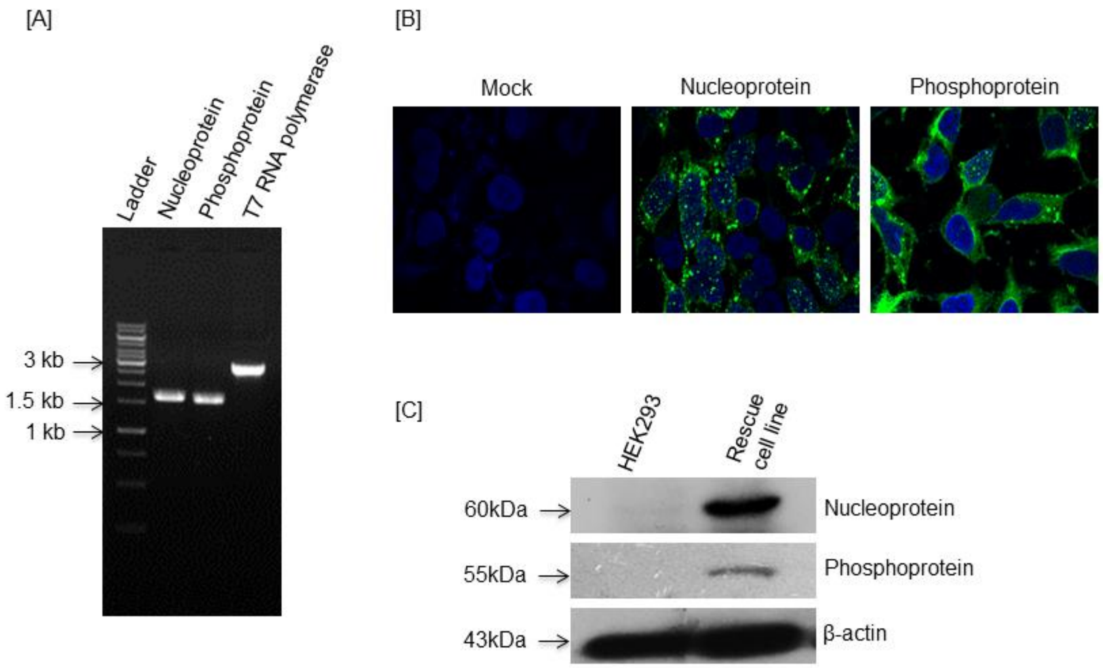

Figure S1. Confirmation of measles virus gene expression in rescue cell line.
Genes encoding measles virus Nucleoprotein (N), Phosphoprotein (P) and T7 RNA polymerase (T7) were cloned into eukaryotic expression vector pcDNA3.1(+). HEK293 cells were co-transfected with all three recombinant constructs generated and stable clones were selected. **(A)** Confirmation of N, P and T7 expression at transcript level by RT-PCR, **(B and C)** Expression of N and P protein by IFA staining and western blot analysis respectively.

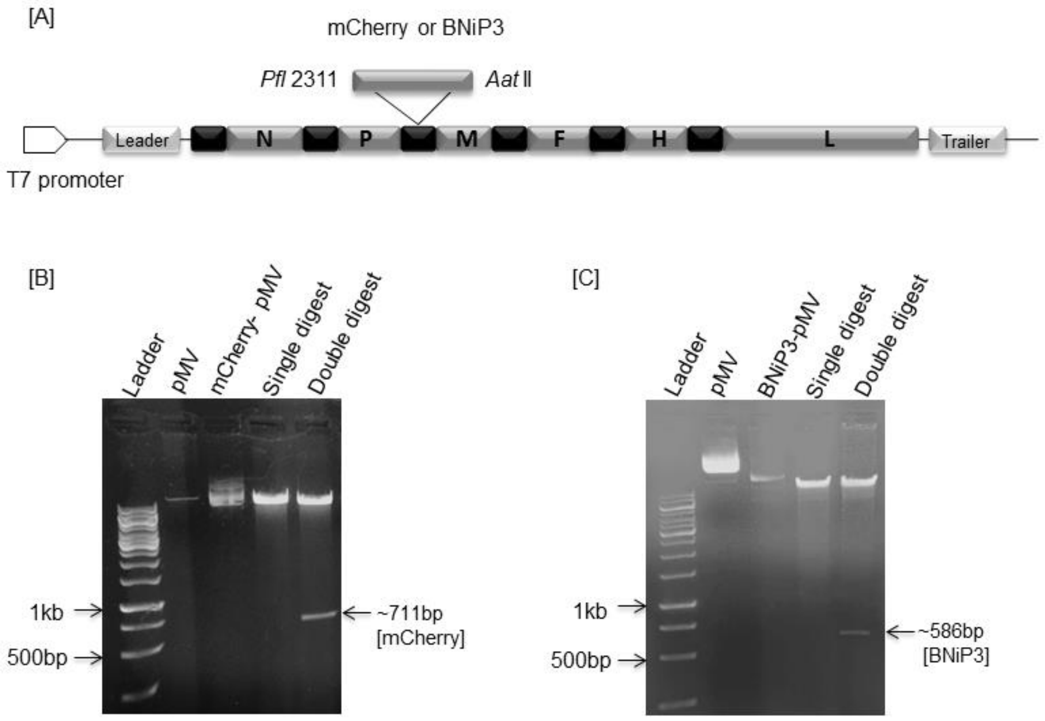

Figure S2. Insertion of mCherry or BNiP3 gene into full length viral genome.
Gene encoding for mCherry reporter protein was amplified from pmCherry-C1 vector. BNiP3, a pro-apoptotic gene was amplified from cells treated with an apoptosis inducer, which enabled the cells to express pro-apoptotic genes. Amplified products were purified and cloned into full length viral genome between P and M protein coding region. **(A)** Position in the viral genome at which foreign gene was inserted. **(B and C)** Fall out of mCherry and BNiP3 clones following restriction digestion.

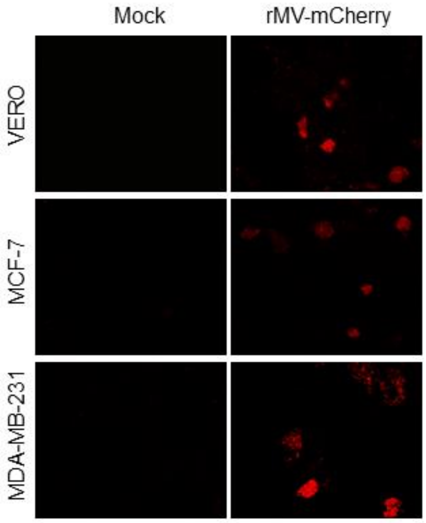

Figure S3. Expression of mCherry in rMV-mCherry infected cells.
Measles virus harboring mCherry gene was rescued from packaging cells following the protocol as described in the text. Vero, MCF-7 and MDA-MB-231 cells were infected with rescued rMV-mCherry recombinant virus and mCherry expression (red fluorescence) was visualized directly under fluorescent microscope.

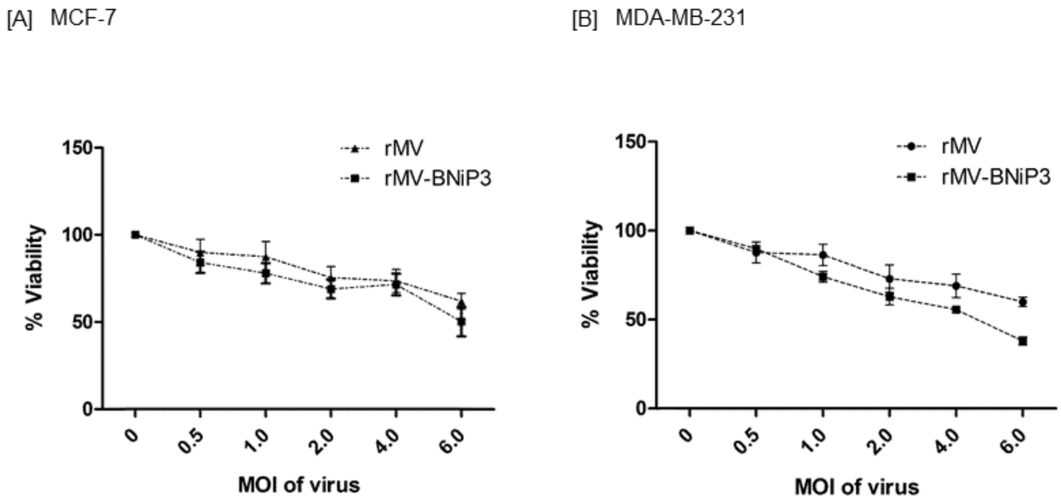

Figure S4. Cytotoxic effects of rMV and rMV-BNiP3 at different multiplicities of infection. MCF-7 and MDA-MB-231 cells were infected with rMV or rMV-BNiP3 at different MOIs (0.5-6.0). Infected cells were incubated at 37ºC; CO_2 and at 56hrs post-infection cells were subjected to MTT assay to measure the cell viability. Percentage of cell viability in **(A)** MCF-7 **(B)** MDA-MB-231 cells infected with rMV or rMV-BNiP3.

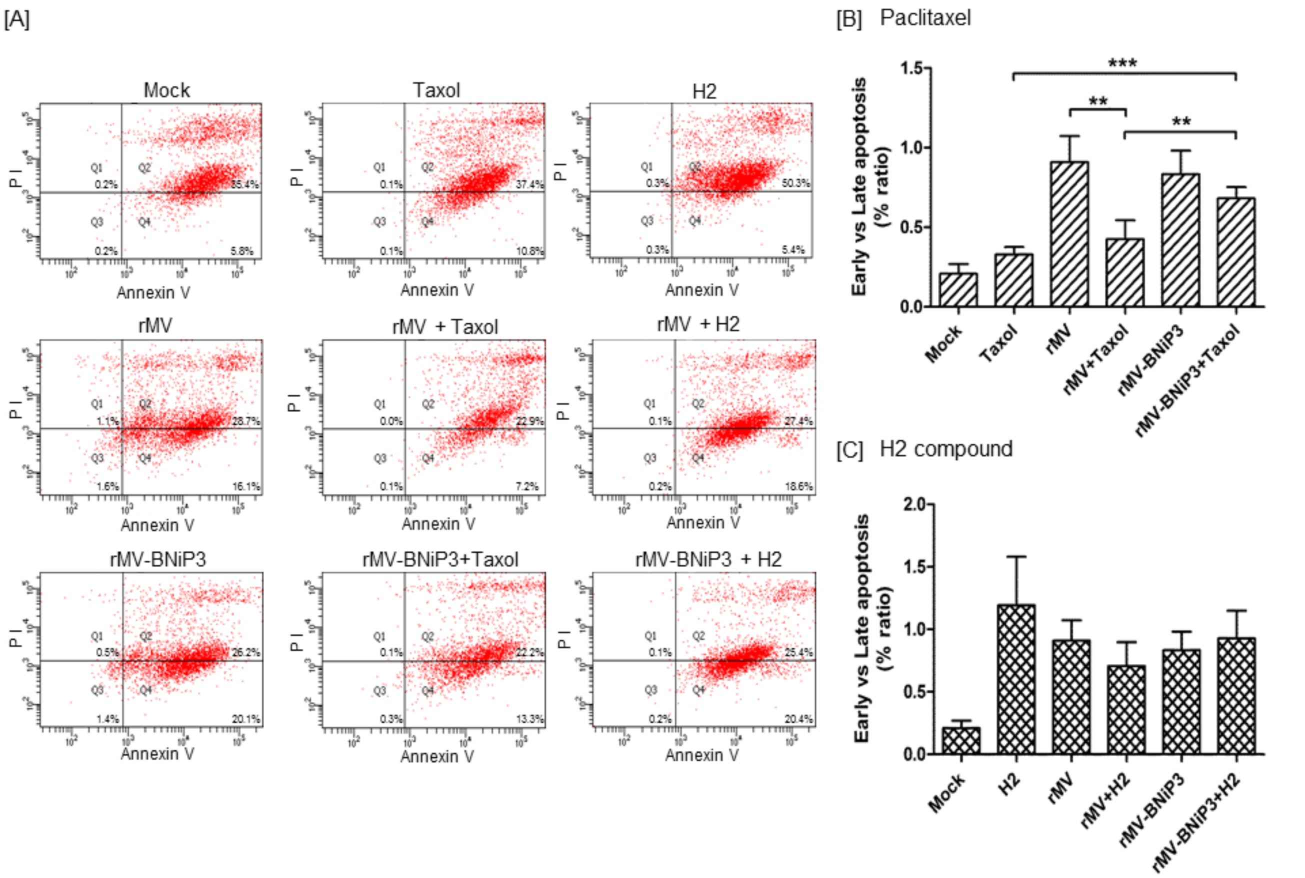

Figure S5. Annexin V staining of MCF-7 cells infected with recombinant virus and treated with drugs.

MCF-7 cells were infected with rMV or rMV-BNiP3 then treated with desired concentration of paclitaxel and H2 compound. Infected cells were subjected to FACS analysis for annexin V staining as described in Figure 7. **(A)** Quadrants representing annexin V and annexin V/PI stained cells. Percentage ratio of Annexin V/PI staining *vs.* Annexin V staining in MCF-7 cells treated with **(B)** paclitaxel **(C)** H2 compound. Population of annexin V positive cells was higher in cells infected with rMV-BNiP3 followed by treatment with paclitaxel (p=0.0002) as compared to paclitaxel alone. Number of annexinV positive cells were higher in cells infected with rMV-BNiP3 followed by treatment with paclitaxel (p=0.0099) as compared to cells treated with combination of rMV and paclitaxel. Cells infected with rMV (p=0.0058) showed higher population of annexin V positive cells as compared to cells treated with the combination of rMV and paclitaxel due to comparably higher population of PI positive dead cells. No such significance was observed in cells treated with the combination of either virus with H2.

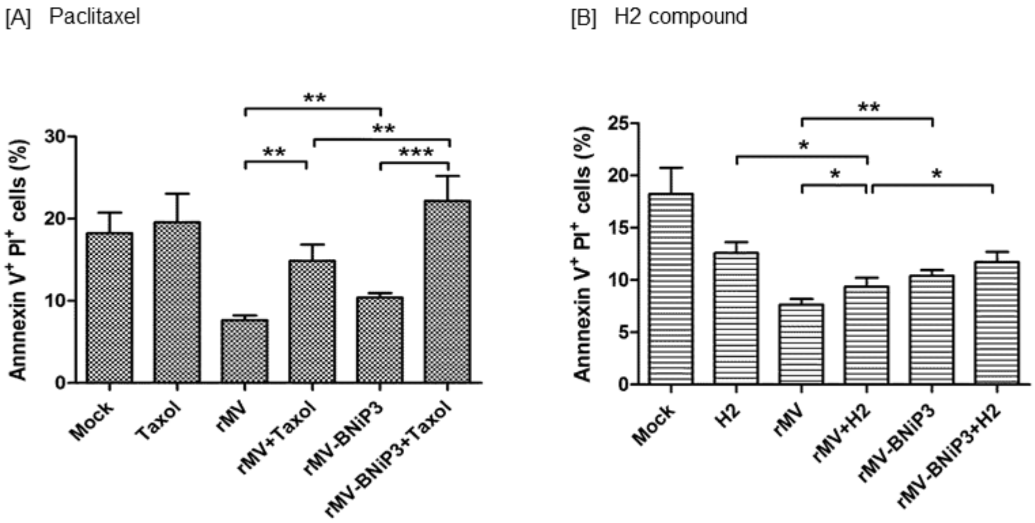

Figure S6. Annexin V and PI positive MDA-MB-231 cells infected with recombinant virus and treated with drugs.

Data presented in Figure 7 is plotted for Q2 quadrant of the scatter plot. The data represented as column graph indicates the population of annexin V and PI positive cells in rMV-BNiP3 infected cells as compared to rMV infected cells or cells treated with drug alone. Percentage of annexin V and PI positive cells treated with **(A)** paclitaxel **(B)** H2 compound. Population of annexin V and PI positive cells were higher in cells infected with rMV-BNiP3 (p=0.0013) as compared to rMV. Cells treated with combination of rMV-BNiP3 with paclitaxel (p=0.0005) as well as rMV with paclitaxel (p=0.0036) showed higher population of annexin V and PI positive cells as compared to respective virus controls. Cells treated with combination of rMV-BNiP3 with paclitaxel (p=0.0246) showed higher population of annexin V and PI positive cells as compared rMV with paclitaxel. Population of annexin V and PI positive cells was higher in cells infected with rMV followed by treatment with H2 as compared to rMV (p=0.0438) alone but lower as compared to H2 (p=0.0144) alone due to comparably higher annexin V positive population. Cells treated with combination of rMV-BNiP3 with H2 (p=0.0211) showed higher population of annexin V and PI positive cells as compared rMV with H2.

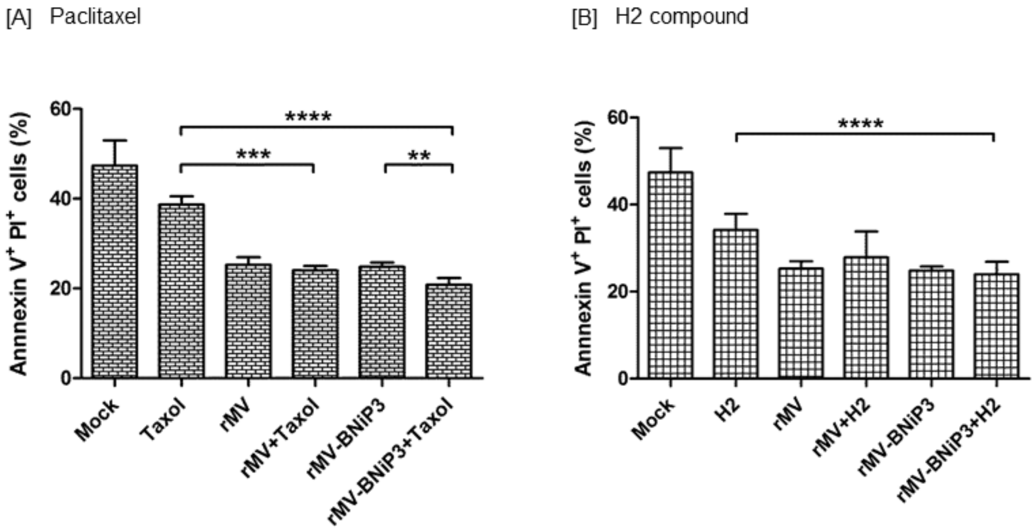

Figure S7. Annexin V and PI positive MCF-7 cells infected with recombinant virus and treated with drugs.

Data presented in Figure S5 is plotted for Q2 quadrant of the scatter plot. The data represented as column graph indicates the population of annexin V and PI positive cells in rMV-BNiP3 infected cells as compared to rMV infected cells or cells treated with drug alone. Percentage of annexin V and PI positive cells treated with **(A)** paclitaxel **(B)** H2 compound. Population of annexin V and PI positive cells were higher in cells treated with paclitaxel as compared to cells infected with either rMV (p=0.0031) or rMV-BNiP3 (p=<0.0001) alone. Cells treated with rMV-BNiP3 (p=0.0039) showed higher population of annexin V and PI positive cells as compared to cells treated with combination of rMV-BNiP3 with paclitaxel. Population of annexin V and PI positive cells was higher in cells treated with H2 (p=0.0093) as compared to cells treated with combination of rMV-BNiP3 and H2.
